# Petroselinic acid purification and its use for the fermentation of new sophorolipids

**DOI:** 10.1186/s13568-016-0199-7

**Published:** 2016-03-31

**Authors:** Elisabeth I. P. Delbeke, Jonas Everaert, Evelien Uitterhaegen, Stijn Verweire, Arno Verlee, Thierry Talou, Wim Soetaert, Inge N. A. Van Bogaert, Christian V. Stevens

**Affiliations:** SynBioC, Department of Sustainable Organic Chemistry and Technology, Ghent University, Coupure Links 653, 9000 Ghent, Belgium; InBio, Department of Biochemical and Microbial Technology, Ghent University, Coupure Links 653, 9000 Ghent, Belgium; Laboratoire de Chimie Agro-industrielle, ENSIACET, Université de Toulouse, INP, 4 Allée Emile Monso, BP 44362, 31030 Toulouse Cedex 4, France

**Keywords:** Petroselinic acid, Sophorolipid, *Starmerella bombicola*, Fermentation, Chemical derivatization

## Abstract

Petroselinic acid, a positional isomer of oleic acid, was isolated from the vegetable oil of *Coriandrum sativum* fruits. This uncommon fatty acid was subsequently used as substrate for sophorolipid fermentation with a *Starmerella bombicola* lactone esterase overexpression (oe *sble*) strain. A petroselinic acid based diacetylated sophorolipid lactone was obtained in high purity without incorporation of de novo synthesized fatty acids such as oleic acid. A total production of 40 g/L was obtained. The petroselinic acid based sophorolipid lactone was subsequently hydrolyzed towards the petroselinic acid based sophorolipid acid. For both compounds, their critical micelle concentration (CMC) and corresponding surface tension were compared to their oleic acid based counterparts. Both petroselinic acid based sophorolipids displayed a much lower CMC value than their oleic acid based counterparts, although their minimal surface tension was the same. Besides, the sophorolipid fermentation product was chemically modified towards a novel C12 sophorolipid aldehyde. This derivative constitutes an interesting building block for further modification towards new-to-nature sophorolipids with high potential for self-assembly applications.

**Electronic supplementary material:**

The online version of this article (doi:10.1186/s13568-016-0199-7) contains supplementary material, which is available to authorized users.

## Introduction

**Scheme 1:**
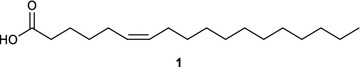
Petroselinic acid

Petroselinic acid **1** is a rather uncommon fatty acid (Scheme 1). With its double bond at the 6, 7-position, it is a positional isomer of oleic acid. The position of this double bond influences the properties of the fatty acid. For example, the melting point of petroselinic acid is 30 °C, while the melting point of oleic acid is only 14 °C (Cahoon et al. 1994). Petroselinic acid is found in high amounts in the seed oils from plants belonging to the *Apiaceae* family, also known as *Umbelliferae*, and the *Araliaceae* family (Placek 1963). The quantity of petroselinic acid varies from 31 to 75 % in the vegetable oil of fruits from *Coriandrum sativum*, one of the most enriched sources of petroselinic acid. This vegetable oil can be extracted from the fruits via twin-screw extrusion (Uitterhaegen et al. 2015). Petroselinic acid **1** is already applied in cosmetic formulations as a moisturizing and anti-aging agent, and as a skin-irritation reducing agent in α-hydroxy acid containing compositions (Alaluf et al. 1999; Barrett et al. 2000; Weinkauf et al. 1998). Besides, a considerable antimicrobial activity against several bacteria, yeast and mold species was observed (Placek 1963). Several modifications of petroselinic acid **1** are described, among others towards surfactants and the nylon 66 precursor adipic acid (Cahoon et al. 1994; Dierker and Schafer 2010; Placek 1963). When incorporated in triglycerides, lipolysis by pancreatic lipase occurs at a much lower efficiency than for oleic acid triglycerides (Cahoon et al. 1994; Heimerma.Wh et al. 1973). Therefore, petroselinic acid rich oils may offer a low-fat alternative for conventional vegetable oils. It was also suggested that petroselinic acid inhibits the synthesis of arachidonic acid, which could counteract the vasoconstrictive effects related to arachidonic acid overproduction (Shukla and Gupta 2009; Weber et al. 1995).

**Scheme 2:**
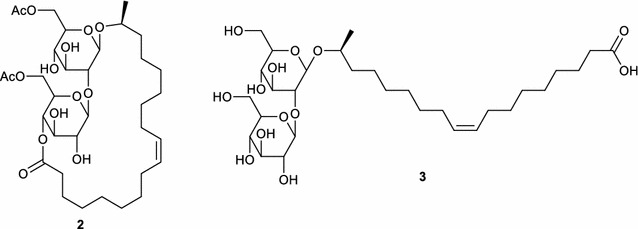
Microbial produced sophorolipid lactone **2** and sophorolipid acid **3**

Being a structural isomer of oleic acid, petroselinic acid could be applied for the synthesis of a new type of sophorolipid. These biosurfactants are produced by different yeast species, mainly *Starmerella bombicola*, from glucose as hydrophilic carbon source and a fatty acid as hydrophobic carbon source (Van Bogaert et al. 2007, 2011c). The main sophorolipid fermentation products are diacetylated C18:1 sophorolipid lactone **2** and C18:1 sophorolipid acid **3** which both include oleic acid in their structure (Scheme 2). Natural sophorolipids feature beneficial biological activities such as anti-cancer, antimicrobial, dermatological, immunoregulatory, spermicidal and antiviral activity (Delbeke et al. 2016; Morya et al. 2013). They also possess self-assembly properties, with a high variety in the type of nanostructures formed for different sophorolipid derivatives (Baccile et al. 2010, 2012, 2013; Cuvier et al. 2014, 2015). For example, C18:1 sophorolipid acid **3** forms micelles with the charge dependent on the pH, while its saturated derivative forms nanoscale ribbons with supramolecular chirality (Cuvier et al. 2014).

**Scheme 3:**
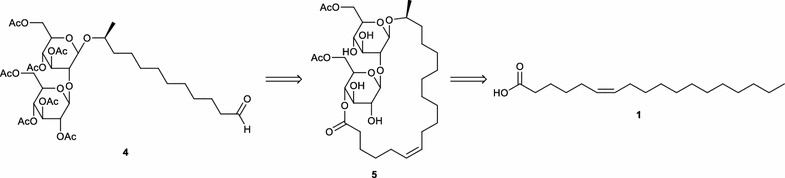
Retrosynthetic scheme for the synthesis of innovative sophorolipid derivatives

As the properties for petroselinic acid differ considerably from those of oleic acid, it can be anticipated that petroselinic acid derived sophorolipids will possess different biological activities and self-assembly properties than the oleic acid based sophorolipids. Moreover, chemical derivatization of these petroselinic acid derived sophorolipids will result in new types of innovative sophorolipid derivatives. In this work, the purification of petroselinic acid **1** and its application for the synthesis of innovative sophorolipid derivatives is described (Scheme 3). First, petroselinic acid is purified from the vegetable oil of *C. sativum* fruits. The purified fatty acid is subsequently used as substrate for sophorolipid fermentation with *S. bombicola*. To obtain 100 % diacetylated sophorolipid lactones, the *S. bombicola* lactone esterase overexpression strain (oe *sble*) is used. Finally, the petroselinic acid derived sophorolipid lactone **5** is modified towards a C12 sophorolipid aldehyde **4** via an ozonolysis reaction.

## Materials and methods

### Materials

The vegetable oil of *C. sativum* fruits was isolated via twin-screw extrusion at LCA via a previously reported procedure (Uitterhaegen et al. 2015). Trimethylsulfonium hydroxide (TMSH), *t*-butyl methyl ether (TBME), hydrochloric acid (HCl), hexane, sodium citrate tribasic dihydrate, ammonium chloride (NH_4_Cl), potassium phosphate monobasic (KH_2_PO_4_), potassium phosphate dibasic (K_2_HPO_4_), magnesium sulfate heptahydrate (MgSO_4_∙7H_2_O), calcium chloride dihydrate (CaCl_2_∙2H_2_O), sodium, 4-dimethylaminopyridine (DMAP), Sudan III and diethyl ether were purchased from Sigma-Aldrich (Belgium). Yeast extract was purchased from Difco (Belgium). Sodium hydroxide (NaOH), dry methanol, acetic anhydride and sodium triacetoxyborohydride [NaBH(OAc)_3_] were purchased from Acros Organics (Belgium). Magnesium sulfate (MgSO_4_) and sodium bicarbonate (NaHCO_3_) were purchased from Fisher Scientific (Belgium). Ethyl acetate and tetrahydrofuran (THF) were purchased from Chem-Lab (Belgium). Glucose monohydrate was purchased from Cargill (Belgium). Sodium chloride (NaCl) was purchased from Colruyt (Belgium).

### Analytical and instrumental methods

Commercially available products were used without further purification. Tetrahydrofuran (THF) was distilled from sodium. The ozonolysis reaction was performed with an Ozonia Triogen Model LAB2B laboratory ozone generator, connected to a Bronkhorst Flow-Bus E-7000 type mass flow meter to control the dry air inflow and an Anseros Ozomat Model GM Non-Dispersive UV-analyzer to measure the ozone concentration. NMR spectra were recorded at 400 MHz (^1^H) and 100 MHz (^13^C) in CDCl_3_ or MeOD with a Bruker Avance III Nanobay 400 MHz spectrometer at room temperature. Low-resolution mass spectra were recorded with a single quadrupole mass spectrometer (ESI, 70 eV). High-resolution mass spectra were obtained with a time-of-flight (TOF) mass spectrometer (ESI or APCI).

### Determination of the fatty acid composition of coriander oil

Determination of the fatty acid composition of the coriander oil was performed by gas chromatography (GC). An oil sample was dissolved in TBME at a concentration of 20 mg/mL. A 100 µL aliquot of this solution was converted to methyl esters by the addition of 50 µL of a 0.2 M TMSH solution in methanol. The resulting fatty acid methyl esters were subjected to GC analysis using a Varian 3800 (USA) gas chromatograph equipped with a flame ionization detector. Separation of the methyl esters was achieved in a CP select CB (Varian, USA) fused silica capillary column (50 m, 0.25 mm i.d., 0.25 µm film thickness). The initial oven temperature was held at 185 °C for 40 min, after which it was increased to 250 °C for 10 min. The temperature of the injector and the detector was kept at 250 °C. Helium was used as the carrier gas with a flow rate of 1.2 mL/min. The determination was carried out in triplicate.

### General procedure for the isolation of petroselinic acid 1

In a 2 L flask, 446.10 g of coriander oil is weighed and 800 mL of a 3 N sodium hydroxide solution is added. The reaction mixture is refluxed for 2.5 h, cooled down and acidified to pH 1 with a 3 N hydrochloric acid solution. The mixture is extracted with hexane and washed with water. The organic phase is dried over magnesium sulfate, filtered and concentrated under reduced pressure. Pure petroselinic acid is obtained through crystallization in absolute ethanol at −20 °C as a white solid at room temperature (251.07 g, 80 %) (Additional file 1: Figs. S1, S2).

### Culture conditions used for sophorolipid production

A *S. bombicola* lactone esterase overexpression strain (oe *sble,* derived from *S. bombicola* ATCC 22214 and covered under the patent application WO 2013/092421) was used for the selective synthesis of diacetylated sophorolipid lactone **5** (Roelants et al. 2015; Van Bogaert et al. 2011a). The *S. bombicola* oe *sble* strain was cultivated on Lang medium (132 g/L glucose monohydrate; 4 g/L yeast extract; 5 g/L sodium citrate tribasic dihydrate; 1.5 g/L NH_4_Cl; 1 g/L KH_2_PO_4_; 0.16 g/L K_2_HPO_4_; 0.7 g/L MgSO_4_∙7H_2_O; 0.5 g/L NaCl; 0.27 g/L CaCl_2_∙2H_2_O) in a Biostat^®^ B 3 L culture vessel (Sartorius-BBI Systems) with a working volume of 1.1 L. Temperature (30 °C), pH (3.5), stirring rate, and air flow rate (1.5 L/min) were controlled by the Biostat^®^ B control unit (Lang et al. 2000). 100 mL of 30 h old shake flask cultures was used for inoculation of the culture vessel. 20 g of petroselinic acid was added to the reactor just after inoculation. From then on, an extra portion of 5 g petroselinic acid was added every 24 h. The initial pH of 5.8 was allowed to drop spontaneously till 3.5 and was maintained at this value afterwards by automated addition of a 5 N NaOH solution. Additional glucose was added 147 h after inoculation. The stirring rate was set at 600 rpm just after inoculation. After 18 h, the stirring rate was raised to 700 rpm since the dissolved oxygen (pO_2_) dropped to 3 %. After 163 h, the stirring rate was raised once more to 800 rpm since the produced sophorolipid lactones formed a highly viscous broth. Growth of the culture was frequently monitored by measuring optical density at 600 nm (Jasco, V-630 Bio Spectrophotometer). Cell dry weight was obtained by centrifugation (5 min, 14000 rpm, Sigma 4–15 centrifuge) of 1 mL reactor broth in pre-dried and weighed falcons. The pellets were subsequently washed once with 1 mL physiological solution (9 g/L NaCl) and dried at 60 °C to a constant weight. Glucose concentrations were measured with a 2700 Select Biochemistry Analyzer (YSI).

### Sophorolipid purification

The fermentation broth was transferred to a 2 L flask and the culture vessel was rinsed with 500 mL water which was also collected. The fermentation broth was kept overnight at 50 °C to induce precipitation of the sophorolipid lactones. Afterwards, the upper water layer which contains the yeast cells was removed, filtered over a Whatman filter and once more kept overnight at 50 °C to induce precipitation of residual sophorolipid lactones. The sophorolipid fraction was resuspended in water, transferred to a 2 L erlenmeyer and shaken overnight at 4 °C to induce crystallization of the sophorolipid lactones. However, crystallization did not occur as is the case for oleic acid based sophorolipid lactones. Therefore, the dense sophorolipid phase was separated from the water phase, dissolved in ethyl acetate and washed with an aqueous sodium bicarbonate solution. The ethyl acetate phase was dried over magnesium sulfate and concentrated under reduced pressure. The water phase was also extracted with ethyl acetate, washed with sodium bicarbonate, dried over magnesium sulfate and concentrated under reduced pressure (Additional file 1: Figs. S3, S4).

### General procedure for the synthesis of sophorolipid acid 7

In a 100 mL round-bottomed flask, 1.01 g lactonic SL **5** (1.47 mmol, 1 eq) was dissolved in aqueous 3 N NaOH and refluxed for 20 min. The reaction mixture was cooled down and acidified with concentrated HCl to pH 5 to induce precipitation of sophorolipid acid **7** as a white powder. The precipitate was filtered, washed with water and dried under reduced pressure (0.87 g, yield 95 %) (Additional file 1: Figs. S5, S6).

### Surface tension measurements

The determination of the surface tension was performed at room temperature via the Wilhelmy plate method. All glassware and the platinum plate were rinsed with sulfochromic acid prior to the experiment to avoid interference of traces of residual compounds. A dilution series in distilled water ranging from 2.0 g/L to 0.01 mg/L was prepared and the surface tension was determined for each sample. The CMC values were determined as the concentration at which the minimal surface tension was reached when plotting the natural logarithm of the sophorolipid concentration against the surface tension. For sophorolipid lactone **5**, the concentration of solubilized sophorolipid was determined with the TOC-5000 (Shimadzu) since this compound is not well soluble in water. With the bruto formula (C_34_H_56_O_14_), the actual sophorolipid concentration could be calculated.

### General procedure for the synthesis of sophorolipid methyl ester 8

In a 100 mL flame dried round-bottomed flask, sodium methoxide was formed in situ by addition of 0.05 g sodium (2.05 mmol, 0.15 eq) to 20 mL dry methanol and 9.39 g lactonic SL **5** (13.64 mmol, 1 eq) was subsequently added. The flask was equipped with a reflux condenser and a CaCl_2_ guard-tube to protect the reaction mixture from atmospheric moisture. The reaction mixture was stirred for 3 h at reflux temperature, cooled down to room temperature and acidified to neutral pH with acetic acid. The mixture was concentrated under reduced pressure, dissolved in deionized water and cooled down to 0 °C in an ice bath. The sophorolipid methyl ester **8** precipitated as a white powder. The precipitate was filtered, washed with water and dried under reduced pressure (7.81 g, yield 90 %) (Additional file 1: Figs. S7, S8).

### General procedure for the synthesis of peracetylated sophorolipid methyl ester 9

In a 100 mL flame dried round-bottomed flask, 5.59 g sophorolipid methyl ester **8** (8.78 mmol, 1 eq) was dissolved in 50 mL dry THF. The flask was equipped with a CaCl_2_ guard-tube to protect the reaction mixture from atmospheric moisture and 5.93 mL acetic anhydride (61.87 mmol, 7.05 eq) and 0.43 g DMAP (3.51 mmol, 0.4 eq) were added. The reaction mixture was stirred for 1 h at room temperature, concentrated under reduced pressure and dissolved in ethyl acetate. The mixture was washed 3 times with a 10 mL saturated NaHCO_3_-solution and the organic phase was dried over MgSO_4_, filtered and concentrated under reduced pressure. Peracetylated sophorolipid methyl ester **9** was isolated as a viscous colourless oil (8.17 g, quantitative yield) (Additional file 1: Figs. S9, S10).

### General procedure for the synthesis of sophorolipid aldehyde 4

In a 250 mL washing flask, 1.00 g peracetylated sophorolipid methyl ester (1.07 mmol, 1 eq) **9** was dissolved in 50 mL MeOH. 0.09 g NaHCO_3_ (1.07 mmol, 1 eq) and a pinch of Sudan III indicator were added. The reaction mixture was sparged with ozone at room temperature until the red color of the reaction mixture dissipated. After addition of 0.23 g NaBH(OAc)_3_ (1.07 mmol, 1 eq), the mixture was stirred for 1 h at room temperature, concentrated under reduced pressure and dissolved in ethyl acetate. The mixture was washed 3 times with 10 mL brine and the organic phase was dried over MgSO_4_, filtered and concentrated under reduced pressure. Peracetylated sophorolipid aldehyde **4** was purified by automated column chromatography as a viscous colourless oil with a hexane/diethyl ether mixture as eluent (0.60 g, 60 %) (Additional file 1: Figs. S11, S12). Gradient: 2 CV 20 % Et_2_O, 10 CV 20-100 % Et_2_O, 9 CV 100 % Et_2_O.

## Results

### Purification of petroselinic acid

**Scheme 4:**
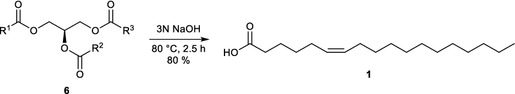
Alkaline hydrolysis towards petroselinic acid

The identification of the different fatty acids and their distribution in the vegetable oil was determined via gas chromatography analysis of the fatty acid methyl esters (Table 1). Petroselinic acid is clearly the major fatty acid, constituting 73.3 % of all fatty acids. Linoleic acid and oleic acid are present in a lower amount, respectively 13.9 and 5 % of all fatty acids. The triglycerides from the vegetable oil were hydrolyzed into glycerol and a fatty acid mixture via an alkaline hydrolysis with sodium hydroxide (Scheme 4). Petroselinic acid **1** was isolated from the reaction mixture via crystallization in absolute ethanol. A high yield of 80 % was obtained, based on the amount of petroselinic acid **1** present in the vegetable oil.

**Table 1:** Fatty acid composition (%) of the vegetable oil of *Coriandrium sativum* fruits

Fatty acid	Content (%)
Palmitic acid (C16:0)	3.2
Palmitoleic acid (C16:1)	0.2
Stearic acid (C18:0)	0.8
Petroselinic acid (C18:1n–12)	72.3
Oleic acid (C18:1n–9)	5.9
cis-Vaccenic acid (C18:1n–7)	1.3
Linoleic acid (C18:2)	13.3
Linolenic acid (C18:3)	0.2
Arachidic acid (C20:0)	0.1
SFA	4.1
MUFA	79.7
PUFA	13.5
Identified fatty acids	97.3

*SFA* saturated fatty acid, *MUFA* monounsaturated fatty acid, *PUFA* polyunsaturated fatty acid, *n.d.* not detected

### Production and characterization of petroselinic acid based sophorolipids

**Fig. 1:**
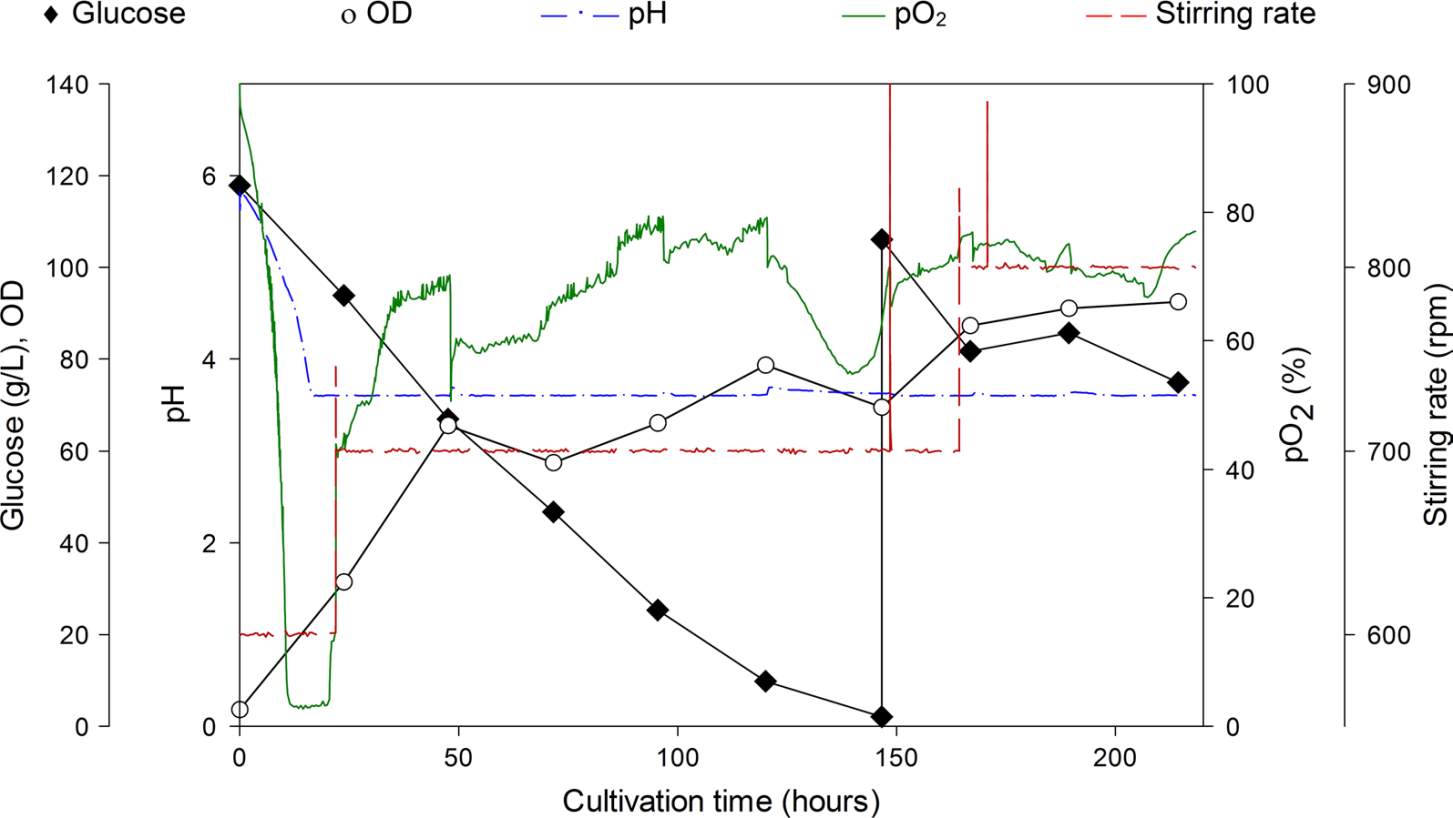
Sophorolipid fermentation parameters depicted in function of time

**Fig. 2:**
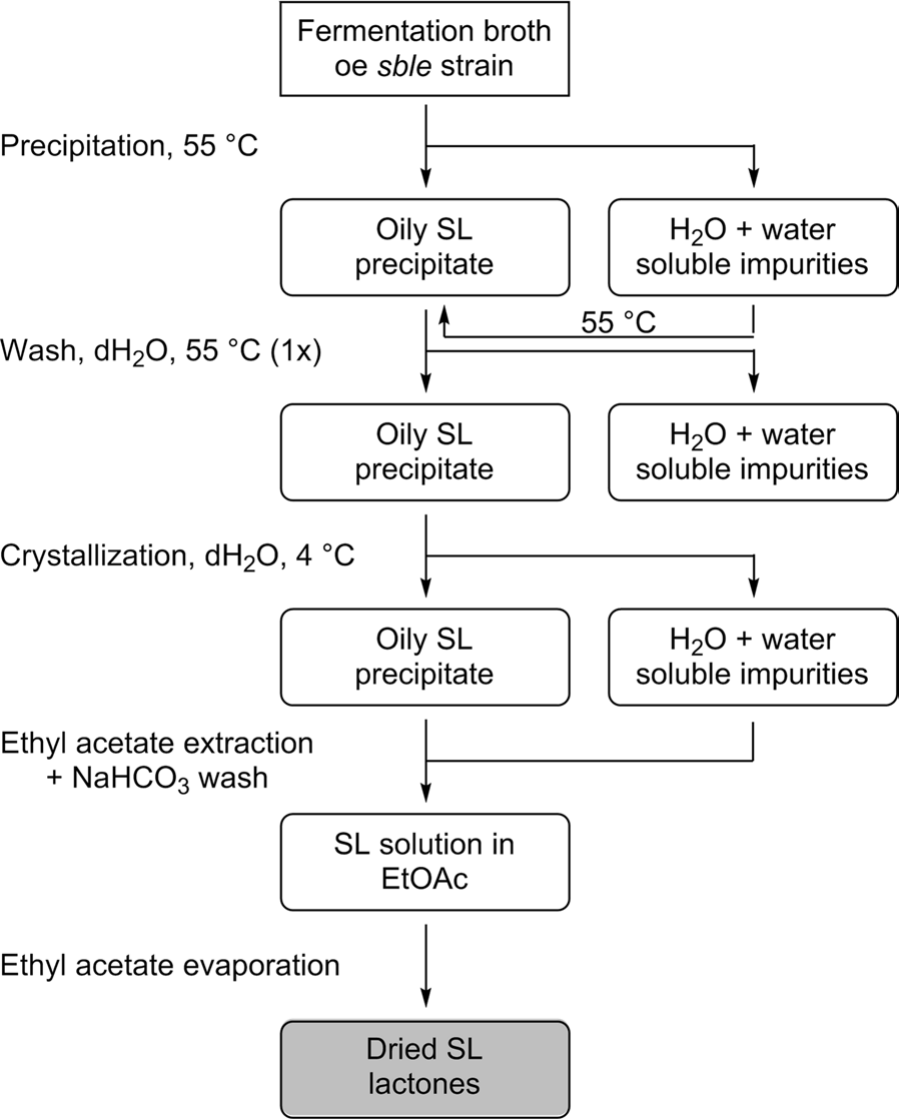
DSP for sophorolipid purification

**Fig. 3:**
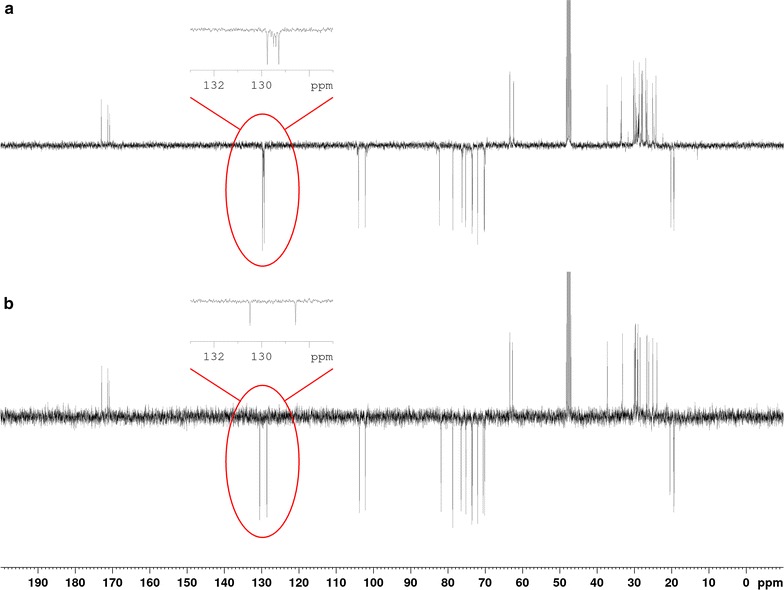
Comparison of sophorolipid lactones by ^13^C-NMR. **a** Oleic acid based sophorolipid lactone, **b** petroselinic acid based sophorolipid lactone

**Scheme 5:**
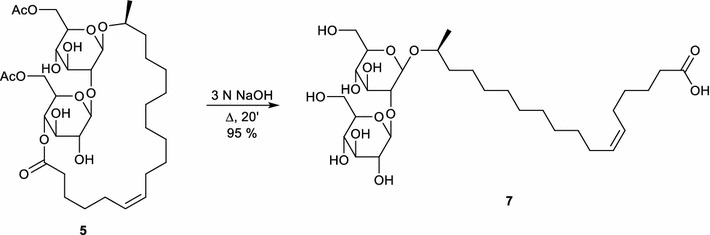
Alkaline hydrolysis towards petroselinic acid based sophorolipid acid **7**

Petroselinic acid **1** was then used as substrate for microbial sophorolipid production (Fig. 1). After the downstream processing, a total amount of 44 g was obtained as a white powder from the combined ethyl acetate fractions (Fig. 2). This corresponds to a total production of 40 g/L. Incorporation of de novo synthesized fatty acids such as oleic acid was not observed via NMR-analysis. Comparison of oleic acid based sophorolipid lactone **2** and petroselinic acid based sophorolipid lactone **5** via ^13^C-NMR clearly demonstrates the difference between the two sophorolipid compounds (Fig. 3). Diacetylated sophorolipid lactone **5** was subsequently subjected to alkaline hydrolysis with NaOH to yield petroselinic acid based sophorolipid acid **7** (Scheme 5).

For both petroselinic acid based sophorolipid compounds, the CMC value and the corresponding surface tension were determined and compared to their oleic acid based counterparts (Table 2). Much lower CMC values are obtained for the petroselinic acid based sophorolipids compared to their oleic acid based counterparts (Roelants et al. 2015). However, the minimal surface tension at these CMC values is almost the same. A less compact geometry for the petroselinic acid based sophorolipids could explain their higher CMC values compared to their oleic acid based counterparts. The increasing hydrophobicity for petroselinic acid based sophorolipid lactone **5** compared to petroselinic acid based sophorolipid acid **7** resulted in a decreasing CMC value, as was the case for their oleic acid based counterparts.

**Table 2:** Comparison of CMC value and corresponding surface tension for petroselinic acid (PA) and oleic acid (OA) based sophorolipid compounds

	CMC (mg/L)	Surface tension (mN/m)
PA SL lactone	4.2 ± 0.1	34.3 ± 0.0
OA SL lactone	45.1 ± 0.1	33.9 ± 0.7
PA SL acid	154 ± 5	42.0 ± 0.3
OA SL acid	245 ± 9	40.9 ± 0.3

The data for the OA based sophorolipid compounds were previously determined by Roelants et al. (2015)

### Chemical modification towards a C12 sophorolipid aldehyde

**Scheme 6:**
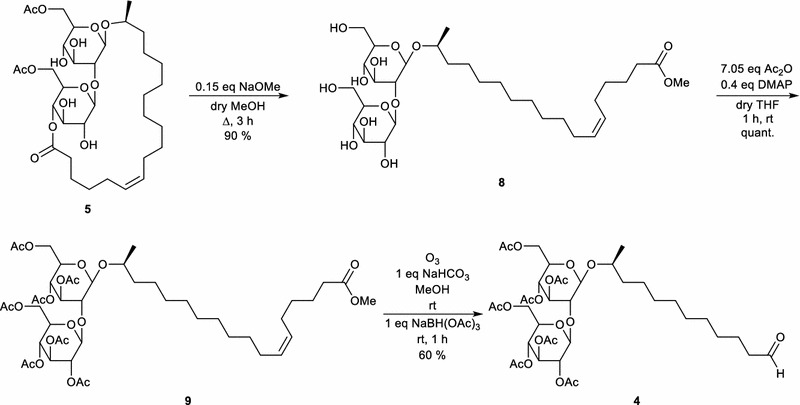
Chemical modification towards sophorolipid aldehyde **4**

Diacetylated sophorolipid lactone **5** was used for the synthesis of mid-chain sophorolipid aldehyde **4** (Scheme 6). A chemical modification pathway towards sophorolipid aldehydes starting from the microbially produced sophorolipid lactones was already developed in our laboratory for oleic acid derived sophorolipids (Delbeke et al. 2015). In a first step, sophorolipid lactone **5** was transformed into sophorolipid methyl ester **8** via an alkaline hydrolysis with sodium methoxide in methanol. Protection of the sugar head group via an acetylation reaction yielded peracetylated sophorolipid methyl ester **9**. Cleavage of the double bond was obtained via an ozonolysis reaction, resulting in the synthesis of sophorolipid aldehyde **4**.

## Discussion

Different hydrophobic substrates other than oleic acid have already been reported for sophorolipid fermentation, for example hydroxylated fatty acids (Van Bogaert et al. 2011b), arachidonic acid (Prabhune et al. 2002; Shah and Prabhune 2007; Van Bogaert et al. 2009), linoleic acid (Kasture et al. 2008) and linolenic acid (Gupta and Prabhune 2012). In this work, a synthetic pathway towards new-to-nature sophorolipids was developed via the incorporation of petroselinic acid **1** in the fermentation pathway. Incorporation of petroselinic acid in sophorolipid derivatives was never described before and offers many advantages. First of all, high amounts of petroselinic acid **1** are present in the vegetable oil of *C. sativum* fruits, making this fatty acid economically interesting as hydrophobic substrate for sophorolipid fermentation. Furthermore, the position of the double bond of petroselinic acid **1** greatly influences the characteristics of this fatty acid as compared to oleic acid. Therefore, it is anticipated that incorporation of petroselinic acid in sophorolipid compounds can also have a great influence on the sophorolipid properties. Besides, synthesis of sophorolipid compounds with the double bond at an alternative position opens possibilities for the synthesis of mid-chain C12 sophorolipid derivatives via an ozonolysis reaction.

Petroselinic acid **1** was isolated from the vegetable oil of *C. sativum* fruits via twin-screw extrusion and alkaline hydrolysis in a high yield of 80 % and was subsequently used as substrate for sophorolipid fermentation with a *S. bombicola* lactone esterase overexpression strain (oe *sble*). This modified strain only produces sophorolipid lactones, thus resulting in a more homogenous sophorolipid product than the one that can be obtained with the *S. bombicola* wild type. The desired sophorolipid lactone **5** was obtained in high purity without incorporation of *de novo* synthesized fatty acids. The corresponding petroselinic acid based sophorolipid acid **7** was synthesized from sophorolipid lactone **5** via alkaline hydrolysis. Petroselinic acid based sophorolipid lactone **5** and acid **7** have a much lower CMC value than their oleic acid based counterparts, which indicates that the fatty acid incorporated in the sophorolipid has a great influence on the geometric structure of the derivatives. The sophorolipid fermentation product was chemically modified towards C12 sophorolipid aldehyde **4**. This derivative constitutes an interesting building block for further modification towards new-to-nature sophorolipids with high potential for self-assembly applications. Similar modifications as those described for oleic acid based sophorolipids are currently in progress (Delbeke et al. 2015).

## Additional file

10.1186/s13568-016-0199-7 ^1^H-NMR spectrum for compound **1**. **Fig. S2.** ^13^C-NMR spectrum for compound **1**. **Fig. S3.** ^1^H-NMR spectrum for compound **5**. **Fig. S4.** ^13^C-NMR spectrum for compound **5**. **Fig. S5.** ^1^H-NMR spectrum for compound **7**. **Fig. S6.** ^13^C-NMR spectrum for compound **7**. **Fig. S7.** ^1^H-NMR spectrum for compound **8**. **Fig. S8.** ^13^C-NMR spectrum for compound **8**. **Fig. S9.** ^1^H-NMR spectrum for compound **9**. **Fig. S10.** ^13^C-NMR spectrum for compound **9**. **Fig. S11.** ^1^H-NMR spectrum for compound **4**. **Fig. S12.** ^13^C-NMR spectrum for compound **4**.
