# Supplementary material for: Petroselinic acid purification and its use for the fermentation of new sophorolipids
Source: AMB Express. 2016 Mar 31;6:28. doi: 10.1186/s13568-016-0199-7 (PMC4816958; doi:10.1186/s13568-016-0199-7)
Supplement: Supplementary file 1 — 10.1186/s13568-016-0199-7 1H-NMR spectrum for compound 1. Fig. S2. 13C-NMR spectrum for compound 1. Fig. S3. 1H-NMR spectrum for compound 5. Fig. S4. 13C-NMR spectrum for compound 5. Fig. S5. 1H-NMR spectrum for compound 7. Fig. S6. 13C-NMR spectrum for compound 7. Fig. S7. 1H-NMR spectrum for compound 8. Fig. S8. 13C-NMR spectrum for compound 8. Fig. S9. 1H-NMR spectrum for compound 9. Fig. S10. 13C-NMR spectrum for compound 9. Fig. S11. 1H-NMR spectrum for compound 4. Fig. S12. 13C-NMR spectrum for compound 4. [file 13568_2016_199_MOESM1_ESM.pdf]

AMB Express

Electronic Supporting Information

Petroselinic acid purification and its use for the fermentation of new sophorolipids

Elisabeth I.P. Delbeke[a], Jonas Everaert[a,b], Evelien Uitterhaegen[a,c], Stijn Verweire[b], Arno Verlee[a], Thierry Talou[c], Wim Soetaert[b], Inge N.A. Van Bogaert[b] and Christian V. Stevens[a*]

[a] SynBioC, Department of Sustainable Organic Chemistry and Technology, Ghent University, Coupure Links 653, 9000 Ghent, Belgium.

[b] InBio, Department of Biochemical and Microbial Technology, Ghent University, Coupure Links 653, 9000 Ghent, Belgium

[c] Laboratoire de Chimie Agro-industrielle, ENSIACET, Université de Toulouse, INP, 4 Allée Emile Monso, BP 44362, 31030 Toulouse Cedex 4, France.

Elisabeth.Delbeke@UGent.be, Jonas.Everaert@UGent.be, Evelien.Uitterhaegen@ensiacet.fr, Stijn.Verweire@UGent.be, Arno.Verlee@UGent.be, Thierry.Talou@ensiacet.fr, Wim.Soetaert@UGent.be, Inge.VanBogaert@UGent.be

Corresponding author: Chris.Stevens@UGent.be, SynBioC, Department of Sustainable Organic Chemistry and Technology, Ghent University, Coupure Links 653, 9000 Ghent, Belgium.

Characterization data for petroselinic acid (1).

^{1}H-NMR (400 MHz, $CDCl_3$): δ_H 0.88 (3H, t, *J*=6.8 Hz, $C\underline{H}_3$), 1.26-1.36 (18H, m, 9xCH_2), 1.37-1.44 (2H, m, $C\underline{H}_2(CH_2)_2COOH$), 1.62-1.69 (2H, m, $C\underline{H}_2CH_2COOH$), 2.01 (2H, dxt, *J*=7.0 Hz, *J*=6.9 Hz, $C\underline{H}_2CH{=}CH$), 2.05 (2H, dxt, J=7.1 Hz, J=7.1 Hz, $C\underline{H}_2CH{=}CH$), 2.36 (2H, t, *J*=7.5 Hz, $C\underline{H}_2COOH$), 5.29-5.41 (2H, m, $\underline{CH}{=}C\underline{H}$), 11.60 (1H, br s, $COO\underline{H}$). **^{13}C-NMR (100 MHz, $CDCl_3$): δ_C** 14.1 ($\underline{C}H_3$), 22.7 ($\underline{C}H_2$), 24.3 ($\underline{C}H_2CH_2COOH$), 26.8 ($\underline{C}H_2CH{=}CH$), 27.2 ($\underline{C}H_2CH{=}CH$), 29.1 ($\underline{C}H_2(CH_2)_2COOH$), 29.3, 29.4, 29.6, 29.7, 29.7, 29.7, 31.9 (8x$\underline{C}H_2$), 34.0 ($\underline{C}H_2COOH$), 128.9 ($\underline{C}H{=}CH$), 130.6 ($\underline{C}H{=}CH$), 180.1 ($\underline{C}OOH$). **T_m:** 30-31°C.

Characterization data for petroselinic acid based diacetylated sophorolipid lactone (5).

^{1}H-NMR (400 MHz, MeOD): δ_H 1.23 (3H, d, *J*=6.2 Hz, $CHC\underline{H}_3$), 1.31-1.50 (16H, m, 7x$CH_2(CH_2)_2$, $C\underline{H}_aH_bCHCH_3$, $C\underline{H}_aH_bCH_2CHCH_3$), 1.53-1.59 (2H, m, $CH_a\underline{H}_bCHCH_3$, $CH_a\underline{H}_bCH_2CHCH_3$), 1.61-1.74 (2H, m, $C\underline{H}_2CH_2C{=}O$), 2.03-2.11 (4H, m, 2x$C\underline{H}_2CH{=}CH$), 2.06 (3H, s, $C\underline{H}_3C{=}O$), 2.07 (3H, s, $C\underline{H}_3C{=}O$), 2.33-2.47 (2H, m, $C\underline{H}_2C{=}O$), 3.29-3.36 (2H, m, 2x$C\underline{H}OC$), 3.45 (1H, dxd, *J*=9.1 Hz, *J*=7.7 Hz, $C\underline{H}OC$), 3.46-3.50 (1H, m, $C\underline{H}OC$), 3.58 (1H, dxd, *J*=9.1 Hz, *J*=9.1 Hz, $C\underline{H}OC$), 3.60 (1H, dxd, *J*=9.4 Hz, *J*=9.4 Hz, $C\underline{H}OC$), 3.66 (1H, dxdxd, *J*=10.0 Hz, *J*=3.8 Hz, *J*=3.8 Hz, $C\underline{H}OC$), 3.71-3.79 (1H, m, $CH_3C\underline{H}O$), 4.09-4.14 (2H, m, $C\underline{H}_2OAc$), 4.21 (1H, dxd, *J*=11.8 Hz, *J*=6.4 Hz, $C\underline{H}_aH_bOAc$), 4.38 (1H, dxd, *J*=11.8 Hz, *J*=2.1 Hz, $CH_a\underline{H}_bOAc$),4.46 (1H, d, *J*=7.6 Hz, $CH(O)_2$), 4.67 (1H, dxd, *J*=7.7 Hz, $CH(O)_2$), 4.84-4.89 (1H, m, $C\underline{H}OC$), 5.33-5.43 (2H, m, $C\underline{H}{=}C\underline{H}$). **^{13}C-NMR (100 MHz, MeOD): δ_C** 19.3 ($\underline{C}H_3C{=}O$), 19.5 ($\underline{C}H_3C{=}O$), 20.5 ($\underline{C}H_3CH$), 23.9 ($\underline{C}H_2CH_2C{=}O$), 25.1 ($C\underline{H}_2CH_2CHCH_3$), 26.1 ($\underline{C}H_2CH{=}CH$), 26.6 ($\underline{C}H_2CH{=}CH$), 28.5 ($\underline{C}H_2(CH_2)_2$), 29.0 ($\underline{C}H_2(CH_2)_2$), 29.2 ($\underline{C}H_2(CH_2)_2$), 29.7 ($\underline{C}H_2(CH_2)_2$), 29.7 ($\underline{C}H_2(CH_2)_2$), 29.8 ($\underline{C}H_2(CH_2)_2$), 30.0 ($\underline{C}H_2(CH_2)_2$), 33.2 ($\underline{C}H_2C{=}O$), 37.2 ($\underline{C}H_2CHCH_3$), 62.7 ($\underline{C}H_2OAc$), 63.4 ($\underline{C}H_2OAc$), 70.2 ($\underline{C}HOC$), 70.5 ($\underline{C}HOC$), 72.0 ($\underline{C}HOC$), 73.4 ($\underline{C}HOC$), 73.6 ($\underline{C}HOC$), 75.2 ($\underline{C}HOC$), 76.5 ($\underline{C}HOC$), 78.7 ($\underline{C}HOC$), 81.9 ($\underline{C}HOC$), 102.2 ($\underline{C}H(O)_2$), 103.8 ($\underline{C}H(O)_2$), 128.6 ($\underline{C}H{=}CH$), 130.5 ($\underline{C}H{=}CH$), 107.8 ($\underline{C}{=}O$), 171.3 ($\underline{C}{=}O$), 172.9 ($\underline{C}{=}O$).

Characterization data for 17-*L*-([2*β*-*O*-*β*-*D*-glucopyranosyl-*β*-*D*-glucopyranosyl]-oxy)-*cis*-6-octadecenoic acid (7).

^{1}H-NMR (400 MHz, MeOD): δ_H 1.27 (3H, d, *J*=6.2 Hz, $C\underline{H}_3CH$), 1.30-1.48 (17H, m, 8x$CH_2(CH_2)_2$, $C\underline{H}_aH_bCHCH_3$), 1.60-1.67 (3H, m, $CH_a\underline{H}_bCHCH_3$, $C\underline{H}_2CH_2COOH$), 2.04-2.11 (4H, m, 2x$C\underline{H}_2CH{=}CH$), 2.31 (2H, t, *J*=7.4 Hz, $C\underline{H}_2COOH$), 3.47 (1H, dxd, *J*=8.4 Hz, *J*=8.4 Hz, $C\underline{H}OC$), 3.58 (1H, dxd, *J*=8.7 Hz, *J*=8.7 Hz, $C\underline{H}OC$), 3.66-3.71 (2H, m, 2x$C\underline{H}_aH_bOH$), 3.82-3.89 (3H, m, 2x$CH_a\underline{H}_bOH$, $C\underline{H}OC$), 4.47 (1H, d, *J*=7.7 Hz, $C\underline{H}(O)_2$), 4.66 (1H, d, *J*=7.8 Hz, $C\underline{H}(O)_2$), 5.33-5.43 (2H, m, $C\underline{H}{=}C\underline{H}$). **^{13}C-NMR (100 MHz, MeOD): δ_C** 20.5 ($\underline{C}H_3CH$), 24.3 ($\underline{C}H_2CH_2COOH$), 24.9 ($\underline{C}H_2(CH_2)_2$), 26.4 ($\underline{C}H_2CH{=}CH$), 26.8 ($\underline{C}H_2CH{=}CH$), 28.9 ($\underline{C}H_2(CH_2)_2$), 29.0 ($\underline{C}H_2(CH_2)_2$), 29.3 ($\underline{C}H_2(CH_2)_2$), 29.5-29.5 (3x$\underline{C}H_2(CH_2)_2$), 29.6 ($\underline{C}H_2(CH_2)_2$), 33.5 ($\underline{C}H_2COOH$), 36.4 ($\underline{C}H_2CHCH_3$), 3.24-3.36 (5H, m, $C\underline{H}OC$), 3.40 (1H, dxd, *J*=8.7 Hz, *J*=8.7 Hz, $C\underline{H}OC$), 61.3 ($\underline{C}H_2OH$), 61.6 ($\underline{C}H_2OH$), 70.1 ($\underline{C}HOC$), 70.4 ($\underline{C}HOC$), 74.4 ($\underline{C}HOC$), 76.3 (2x$\underline{C}HOC$), 76.8 ($\underline{C}HOC$), 76.9 ($\underline{C}HOC$), 77.5 ($\underline{C}HOC$), 80.5 ($\underline{C}HOC$), 101.3 ($\underline{C}H(O)_2$), 103.2 ($\underline{C}H(O)_2$), 128.9 ($\underline{C}H{=}CH$), 129.9 ($\underline{C}H{=}CH$), 176.2 ($\underline{C}OOH$).

Characterization data for methyl 17-*L*-([2*β*-*O*-*β*-*D*-glucopyranosyl-*β*-*D*-glucopyranosyl]-oxy)-*cis*-6-octadecenoate (8).

^{1}H-NMR (400 MHz, MeOD): δ_H 1.27 (3H, d, *J*=6.2 Hz, $C\underline{H}_3CH$), 1.31-1.50 (17H, m, $C\underline{H}_aH_bCHCH_3$, $8xC\underline{H}_2(CH_2)_2$), 1.60-1.67 (3H, m, $CH_a\underline{H}_bCHCH_3$, $C\underline{H}_2CH_2COOMe$), 2.03-2.10 (4H, m, $2xC\underline{H}_2CH{=}CH$), 2.34 (2H, t, *J*=7.4 Hz, $C\underline{H}_2COOMe$), 3.23-3.36 (5H, m, $C\underline{H}OC$), 3.40 (1H, dxd, *J*=8.9 Hz, *J*=8.9 Hz, $C\underline{H}OC$), 3.45-3.49 (1H, m, $C\underline{H}OC$), 3.58 (1H, dxd, *J*=8.7 Hz, *J*=8.7 Hz, CHOC), 3.65-3.70 (2H, m, $2xC\underline{H}_aH_bOH$), 3.67 (3H, s, $OC\underline{H}_3$), 3.81-3.89 (3H, m, $2xCH_a\underline{H}_bOH$, $C\underline{H}OC$), 4.47 (1H, d, *J*=7.7 Hz, $C\underline{H}(O)_2$), 4.66 (1H, d, *J*=7.8 Hz, $C\underline{H}(O)_2$), 5.32-5.43 (2H, m, $C\underline{H}{=}C\underline{H}$). **^{13}C-NMR (100 MHz, MeOD): δ_C** 20.5 ($\underline{C}H_3CH$), 24.2 ($\underline{C}H_2CH_2COOMe$), 24.9 ($\underline{C}H_2(CH_2)_2$), 26.4 ($\underline{C}H_2CH{=}CH$), 26.8 ($\underline{C}H_2CH{=}CH$), 28.9 ($\underline{C}H_2(CH_2)_2$), 29.0 ($\underline{C}H_2(CH_2)_2$), 29.3 ($\underline{C}H_2(CH_2)_2$), 29.5 ($3x\underline{C}H_2(CH_2)_2$), 29.6 ($\underline{C}H_2(CH_2)_2$), 33.3 ($\underline{C}H_2COOMe$), 36.5 ($\underline{C}H_2CHCH_3$), 50.7 ($O\underline{C}H_3$), 61.4 ($\underline{C}H_2OH$), 61.7 ($\underline{C}H_2OH$), 70.1 ($\underline{C}HOC$), 70.4 ($\underline{C}HOC$), 74.5 ($\underline{C}HOC$), 76.4 ($2x\underline{C}HOC$), 76.8 ($\underline{C}HOC$), 76.9 ($\underline{C}HOC$), 77.5 ($\underline{C}HOC$), 80.5 ($\underline{C}HOC$), 101.3 ($\underline{C}H(O)_2$), 103.3 ($\underline{C}H(O)_2$), 128.9 ($\underline{C}H{=}CH$), 130.0 ($\underline{C}H{=}CH$), 174.5 ($\underline{C}OOMe$).

Characterization data for methyl 17-*L*-([2”,3’,3”,4’,4”,6’,6”-heptaacetoxy-2’-*O*-*β*-*D*-glucopyranosyl-*β*-*D*-glycopyranosyl]-oxy)-cis-6-octadecenoate (9).

^{1}H-NMR (400 MHz, $CDCl_3$): δ_H 1.22 (3H, d, *J*=6.2 Hz, $C\underline{H}_3CH$), 1.26-1.41 (17H, m, $C\underline{H}_aH_bCHCH_3$, $8xC\underline{H}_2(CH_2)_2$), 1.56-1.68 (3H, m, $CH_a\underline{H}_bCHCH_3$, $C\underline{H}_2CH_2COOMe$), 1.98-2.08 (4H, m, $2xC\underline{H}_2CH{=}CH$), 1.98 (3H, s, $C\underline{H}_3C{=}O$), 2.00 (3H, s, $C\underline{H}_3C{=}O$), 2.01 (3H, s, $C\underline{H}_3C{=}O$), 2.03 (3H, s, $C\underline{H}_3C{=}O$), 2.06 (3H, s, $C\underline{H}_3C{=}O$), 2.06 (6H, s, $2xC\underline{H}_3C{=}O$), 2.31 (2H, t, *J*=7.5 Hz, $C\underline{H}_2COOMe$), 3.63-3.75 (4H, m, $2xC\underline{H}CH_2OAc$, $CH_3C\underline{H}O$, $C\underline{H}OC$), 3.67 (3H, s, $OC\underline{H}_3$), 4.06-4.10 (2H, m, $2xC\underline{H}_aH_bOAc$), 4.22-4.31 (2H, m, $2xCH_a\underline{H}_bOAc$), 4.48 (1H, d, *J*=7.6 Hz, $C\underline{H}(O)_2$), 4.73 (1H, d, *J*=8.0 Hz, $C\underline{H}(O)_2$), 4.91 (1H, dxd, *J*=8.9 Hz, *J*=7.8 Hz, $C\underline{H}OC$), 4.93 (1H, dxd, *J*=9.6 Hz, *J*=9.6 Hz, $C\underline{H}OC$), 5.06 (1H, dxd, *J*=9.5 Hz, *J*=9.5 Hz, $C\underline{H}OC$), 5.13 (1H, dxd, *J*=9.3 Hz, *J*=9.3 Hz, $C\underline{H}OC$), 5.16 (1H, dxd, *J*=9.5 Hz, *J*=9.5 Hz, $C\underline{H}OC$), 5.29-5.40 (2H, m, $C\underline{H}{=}C\underline{H}$). **^{13}C-NMR (100 MHz, $CDCl_3$): δ_C** 20.5 ($\underline{C}H_3C{=}O$), 20.5 ($2x\underline{C}H_3C{=}O$), 20.6 ($\underline{C}H_3C{=}O$), 20.7 ($2x\underline{C}H_3C{=}O$), 20.8 ($\underline{C}H_3C{=}O$), 21.2 ($\underline{C}H_3CH$), 24.6 ($\underline{C}H_2CH_2COOMe$), 25.1 ($\underline{C}H_2(CH_2)_2$), 26.8 ($\underline{C}H_2CH{=}CH$), 27.2 ($\underline{C}H_2CH{=}CH$), 29.2 ($\underline{C}H_2(CH_2)_2$), 29.3 ($\underline{C}H_2(CH_2)_2$), 29.6 ($\underline{C}H_2(CH_2)_2$), 29.7 ($\underline{C}H_2(CH_2)_2$), 29.7 ($2x\underline{C}H_2(CH_2)_2$), 29.8 ($\underline{C}H_2(CH_2)_2$), 34.0 ($\underline{C}H_2COOMe$), 36.5 ($\underline{C}H_2CHCH_3$), 51.4 ($O\underline{C}H_3$), 62.0 ($\underline{C}H_2OAc$), 62.2 ($\underline{C}H_2OAc$), 68.2 ($\underline{C}HOC$), 68.9 ($\underline{C}HOC$), 71.3 ($\underline{C}HOC$), 71.7 ($\underline{C}HOC$), 71.8 ($\underline{C}HOC$), 73.1 ($\underline{C}HOC$), 74.6 ($\underline{C}HOC$), 77.7 ($\underline{C}HOC$), 77.9 ($\underline{C}HOC$), 100.4 ($\underline{C}H(O)_2$), 101.1 ($\underline{C}H(O)_2$), 129.0 ($\underline{C}H{=}CH$), 130.5 ($\underline{C}H{=}CH$), 169.2 ($CH_3\underline{C}{=}O$), 169.4 ($CH_3\underline{C}{=}O$), 169.7 ($CH_3\underline{C}{=}O$), 170.0 ($CH_3\underline{C}{=}O$), 170.3 ($CH_3\underline{C}{=}O$), 170.6 ($CH_3\underline{C}{=}O$), 170.6 ($CH_3\underline{C}{=}O$), 174.1 ($\underline{C}OOMe$).

Characterization data for 8-*L*-([2”,3’,3”,4’,4”,6’,6”-heptaacetoxy-2’-*O*-*β*-*D*-glucopyranosyl-*β*-*D*-glucopyranosyl]-oxy)-dodecananal (4).

^{1}H-NMR (400 MHz, $CDCl_3$): δ_H 1.22 (3H, d, *J*=6.2 Hz, $C\underline{H}_3CH$), 1.26-1.43 (13H, m, $6xC\underline{H}_2(CH_2)_2$, $C\underline{H}_aH_bCHCH_3$), 1.55-1.69 (3H, $CH_a\underline{H}_bCHCH_3$, $C\underline{H}_2CH_2(C{=}O)H$), 1.99 (3H, s, $C\underline{H}_3C{=}O$), 2.00 (3H, s, $C\underline{H}_3C{=}O$), 2.01 (3H, s, $C\underline{H}_3C{=}O$), 2.03 (3H, s, $C\underline{H}_3C{=}O$), 2.06 (3H, s, $C\underline{H}_3C{=}O$), 2.08 (6H, s, $2xC\underline{H}_3C{=}O$), 2.42 (2H, txd, *J*=7.4 Hz, *J*=1.9 Hz, $C\underline{H}_2(C{=}O)H$), 3.63-3.74 (4H, m, $2xC\underline{H}CH_2OAc$, $CH_3C\underline{H}O$, $C\underline{H}OC$), 4.06-4.10 (2H, m, $2xC\underline{H}_aH_bOAc$), 4.22-4.31 (2H, m, $2xCH_a\underline{H}_bOAc$), 4.47 (1H, d, *J*=7.6 Hz, $C\underline{H}(O)_2$), 4.72 (1H, d, *J*=8.0 Hz, $C\underline{H}(O)_2$), 4.91 (1H, dxd, *J*=9.3 Hz, *J*=8.4 Hz, $C\underline{H}OC$), 4.93 (1H, dxd, *J*=9.7 Hz, *J*=9.7 Hz, $C\underline{H}OC$), 5.06 (1H, dxd, *J*=9.6 Hz, *J*=9.6 Hz, $C\underline{H}OC$), 5.13 (1H, dxd,

J=9.4 Hz, *J*=9.4 Hz, C$\underline{H}$OC), 5.16 (1H, dxd, *J*=9.5 Hz, *J*=9.5 Hz, C$\underline{H}$OC), 9.76 (1H, t, *J*=1.9 Hz, $\underline{H}$C=O). **^{13}C-NMR (100 MHz, $CDCl_3$): δ_C** 20.5 ($\underline{C}H_3C$=O), 20.5 (2x$\underline{C}H_3C$=O), 20.6 ($\underline{C}H_3C$=O), 20.7 (2x$\underline{C}H_3C$=O), 20.8 ($\underline{C}H_3C$=O), 21.3 ($\underline{C}H_3CH$), 22.1 ($\underline{C}H_2CH_2$(C=O)H), 25.0 ($\underline{C}H_2(CH_2)_2$), 29.1 ($\underline{C}H_2(CH_2)_2$), 29.3 ($\underline{C}H_2(CH_2)_2$), 29.4 ($\underline{C}H_2(CH_2)_2$), 29.7 ($\underline{C}H_2(CH_2)_2$), 29.7 ($\underline{C}H_2(CH_2)_2$), 36.5 ($\underline{C}H_2CHCH_3$), 43.9 ($\underline{C}H_2$(C=O)H), 62.0 ($\underline{C}H_2$OAc), 62.3 ($\underline{C}H_2$OAc), 68.3 ($\underline{C}$HOC), 68.9 ($\underline{C}$HOC), 71.3 ($\underline{C}$HOC), 71.6 ($\underline{C}$HOC), 71.8 ($\underline{C}$HOC), 73.0 ($\underline{C}$HOC), 74.7 ($\underline{C}$HOC), 77.6 ($\underline{C}$HOC), 78.0 ($\underline{C}$HOC), 100.4 ($\underline{C}H(O)_2$), 101.2 ($\underline{C}H(O)_2$), 169.3 ($CH_3\underline{C}$=O), 169.4 ($CH_3\underline{C}$=O), 169.7 ($CH_3\underline{C}$=O), 170.0 ($CH_3\underline{C}$=O), 170.3 ($CH_3\underline{C}$=O), 170.6 ($CH_3\underline{C}$=O), 170.6 ($CH_3\underline{C}$=O), 203.0 (H$\underline{C}$=O).

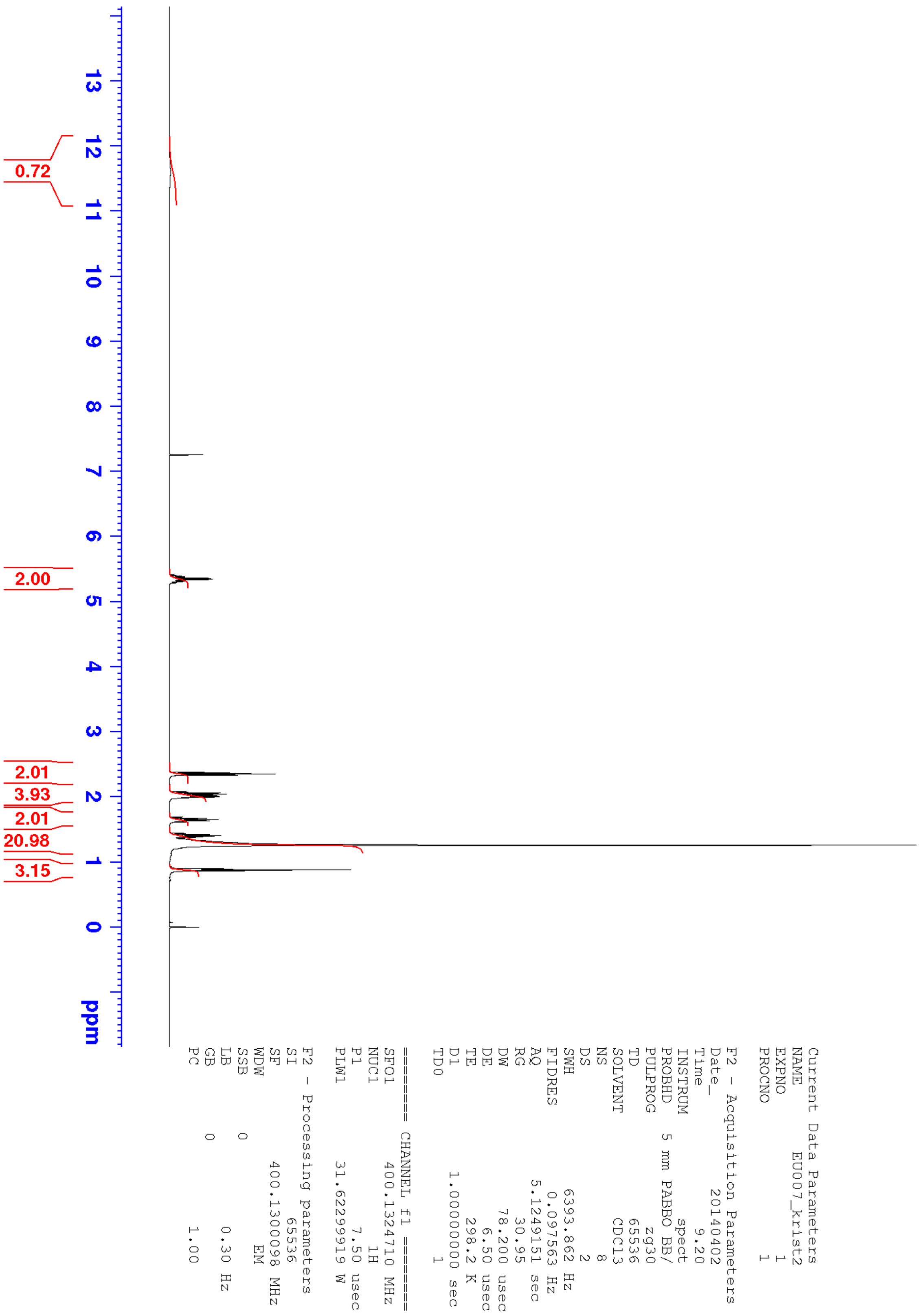

Fig. S1. ^{1}H-NMR spectrum for compound **1**

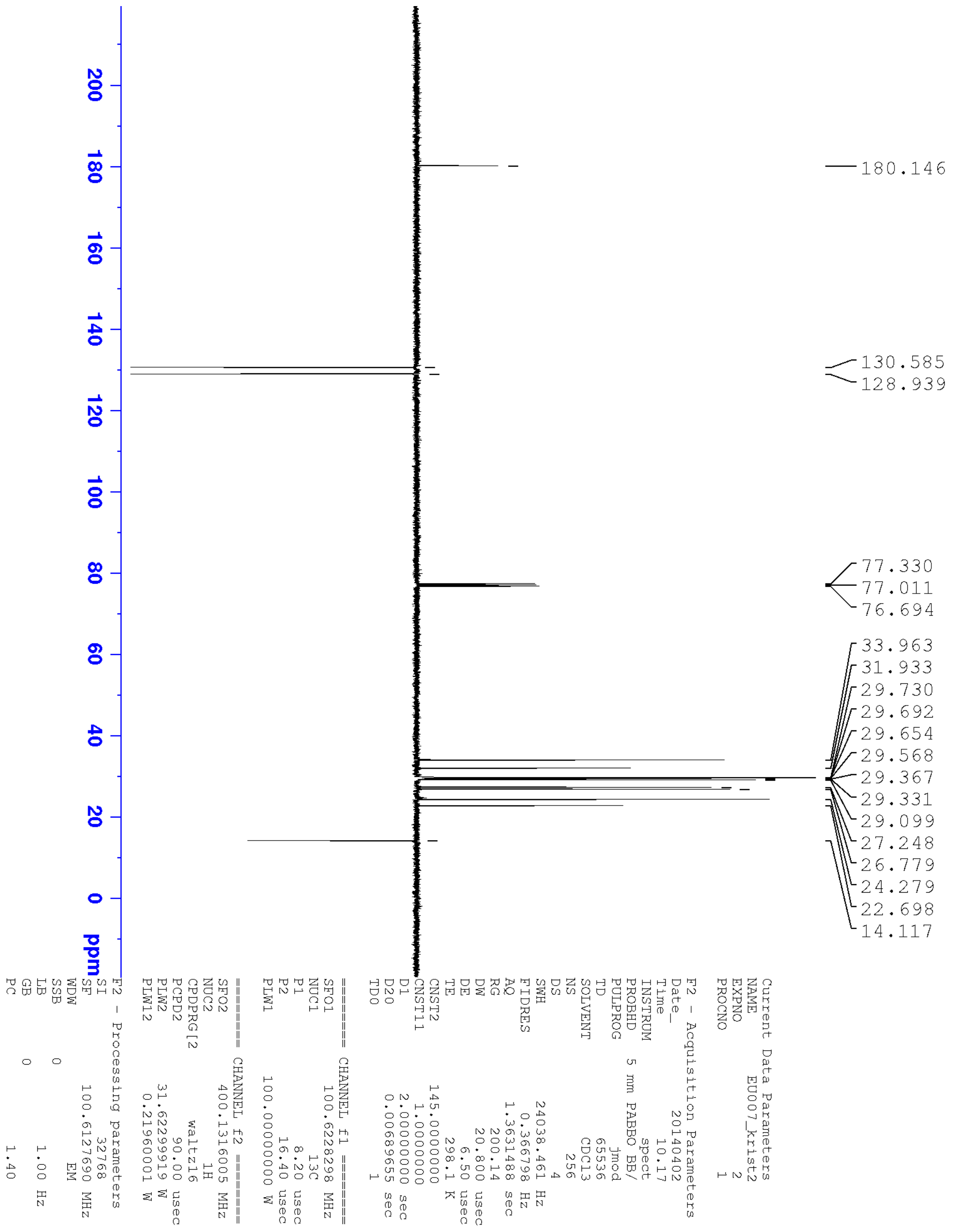

Fig. S2. ^{13}C-NMR spectrum for compound **1**

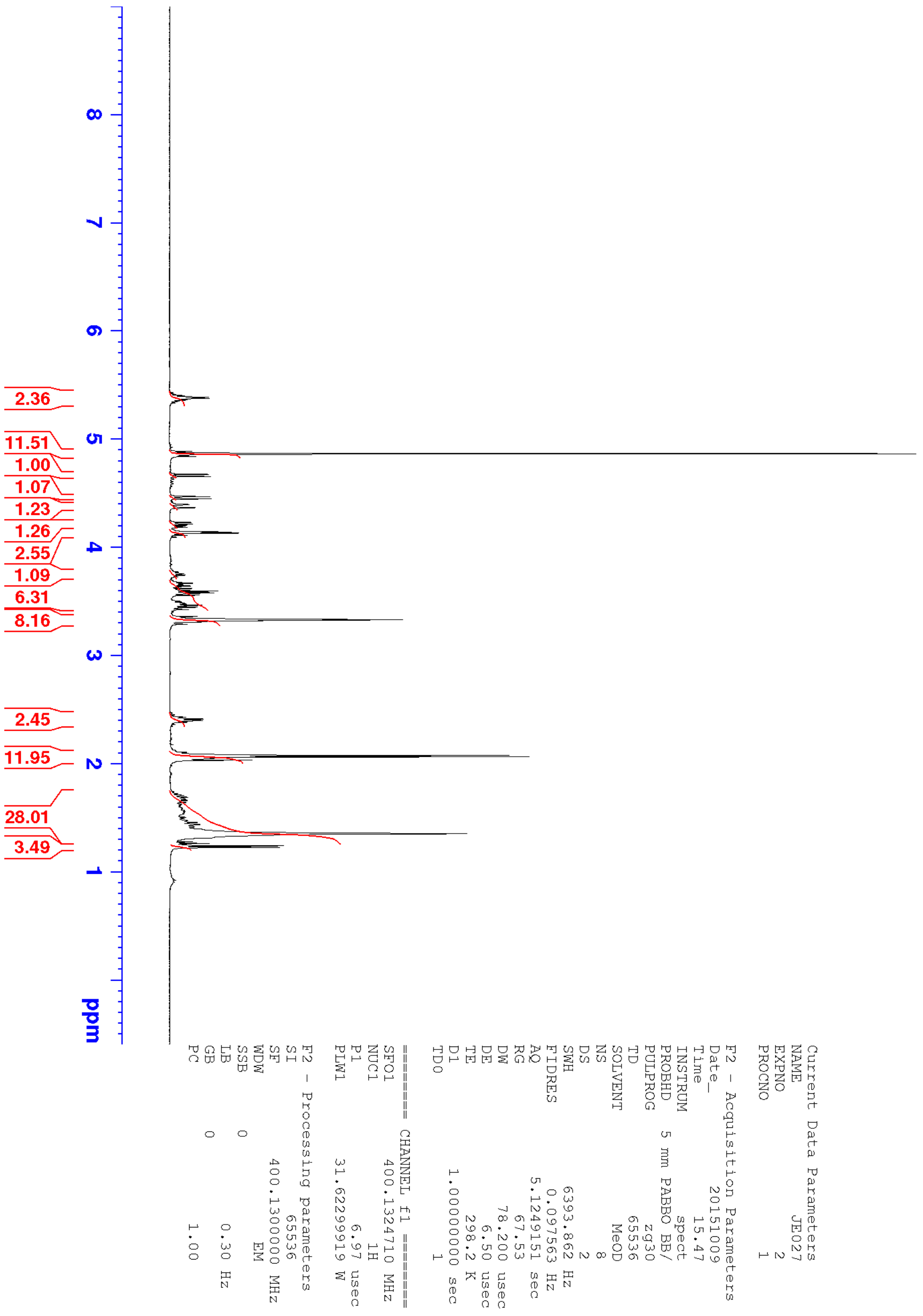

Fig. S3. ^{1}H-NMR spectrum for compound **5**

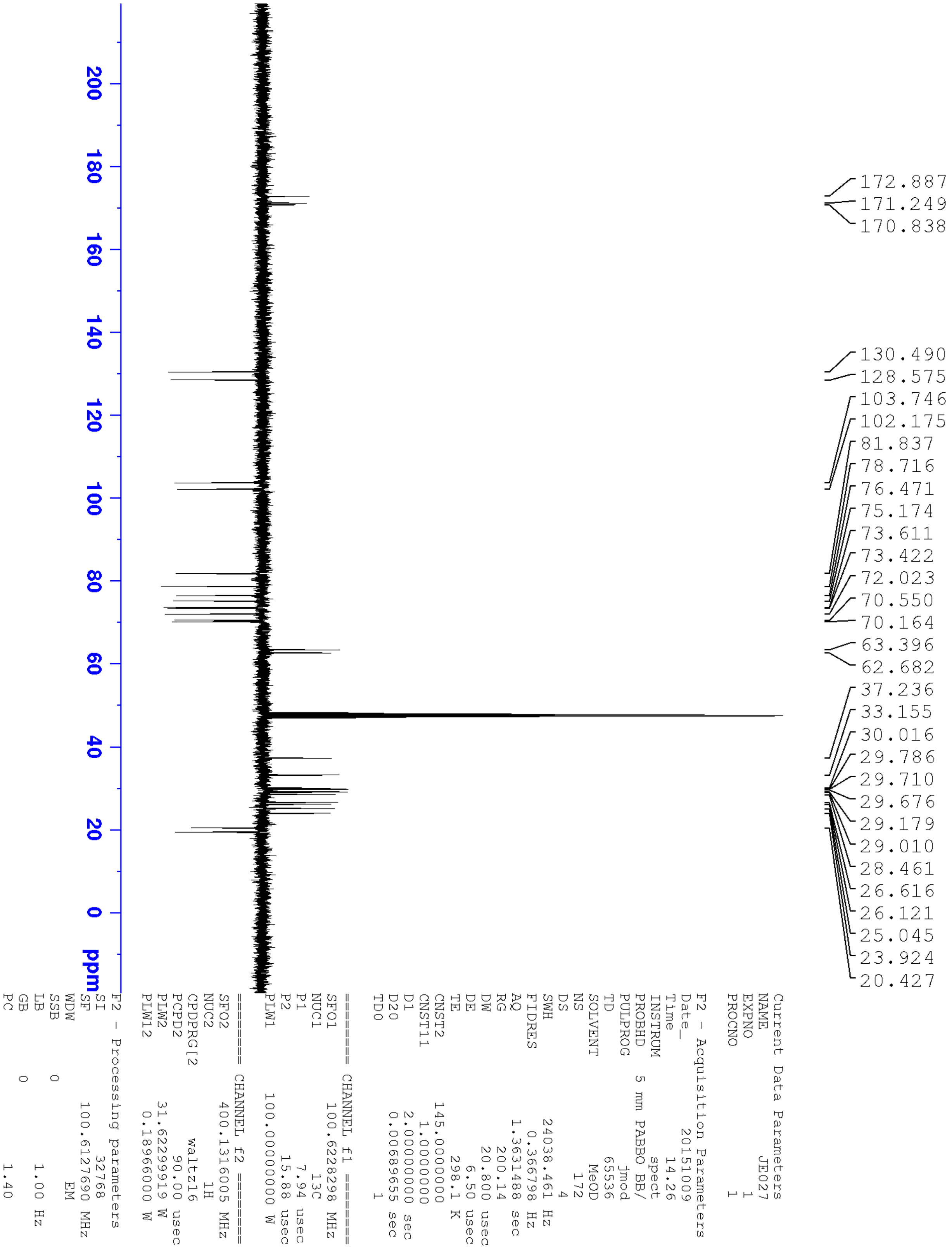

Fig. S4. ^{13}C-NMR spectrum for compound **5**

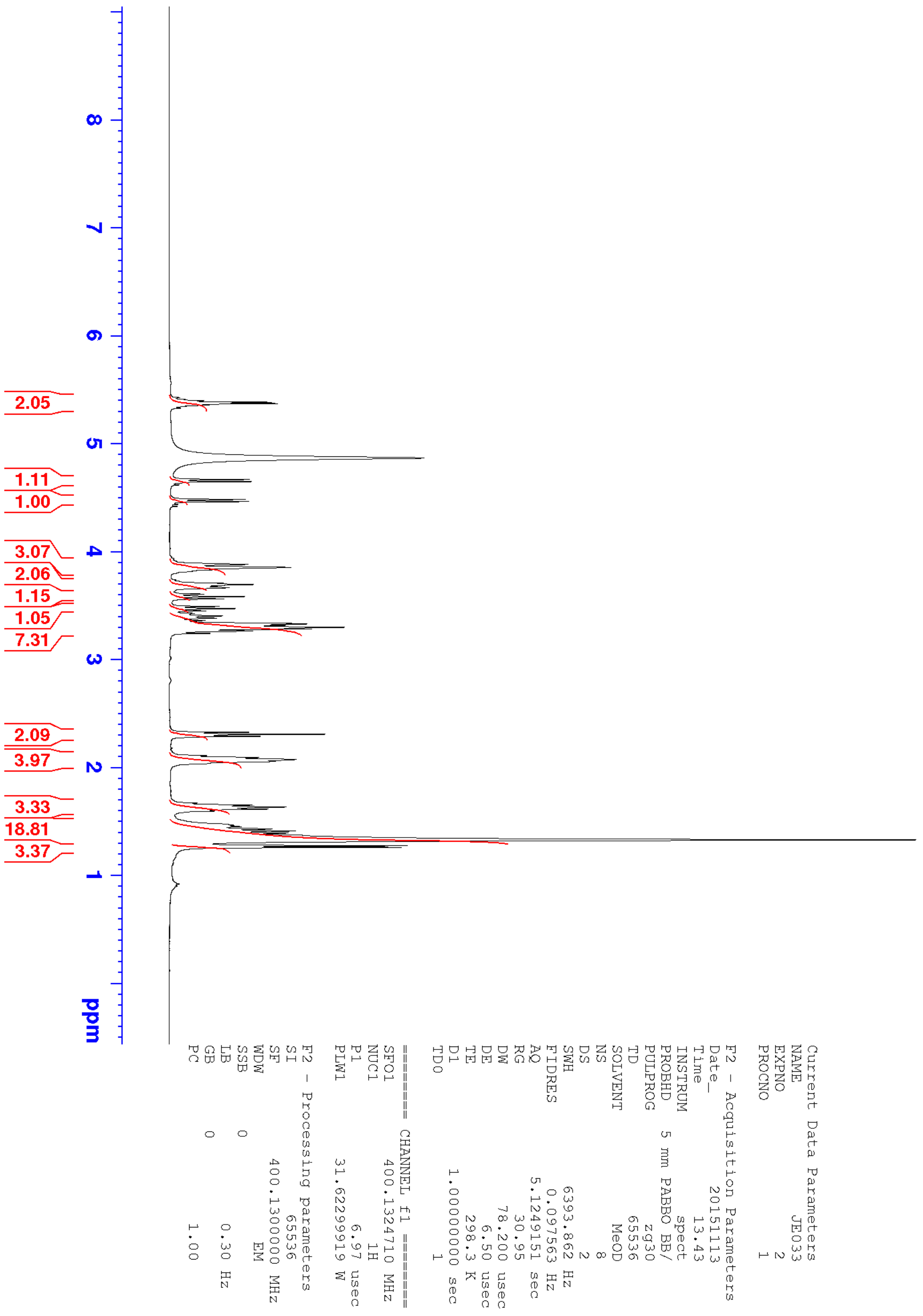

Fig. S5. ^{1}H-NMR spectrum for compound **7**

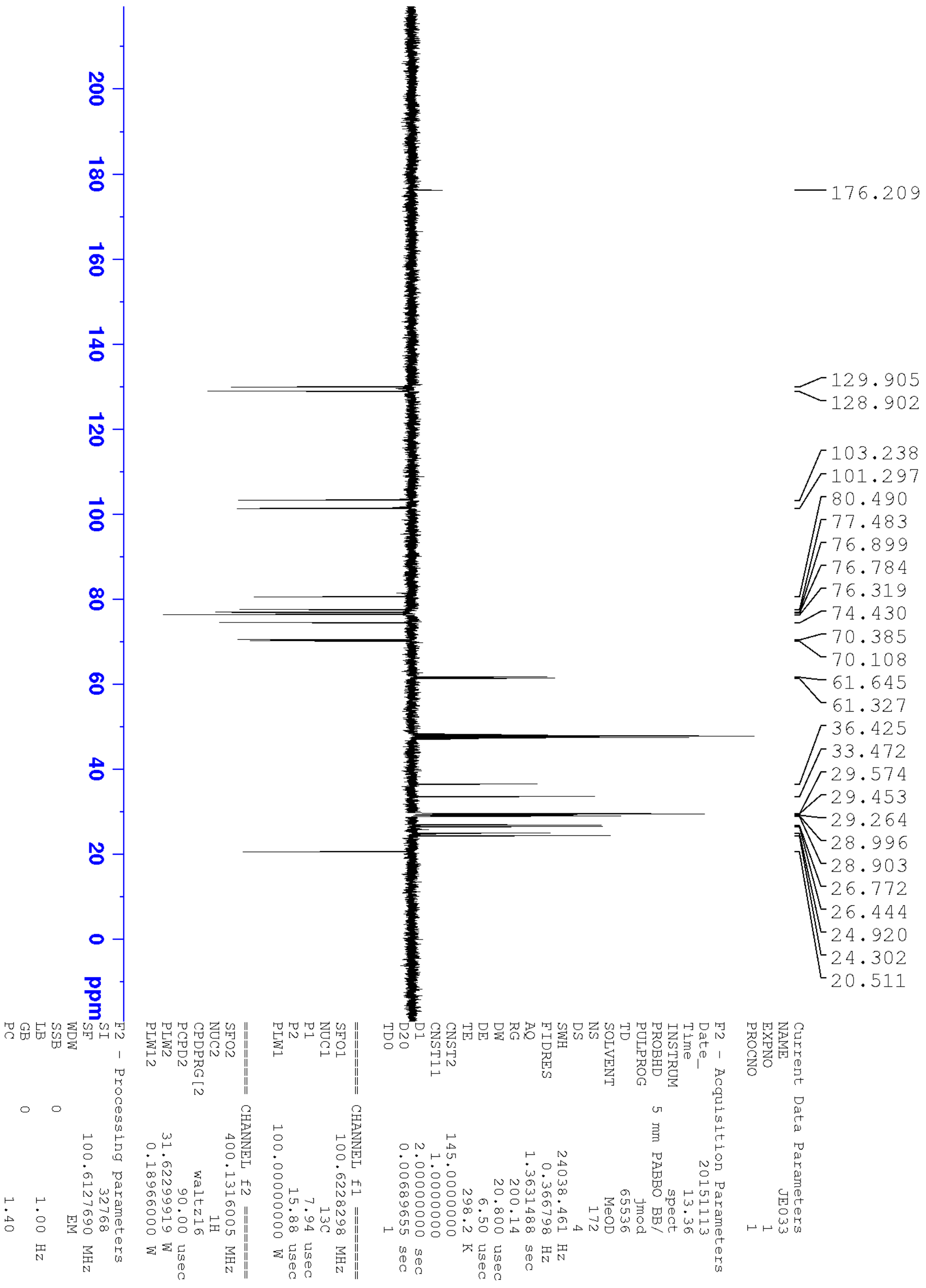

Fig. S6. ^{13}C-NMR spectrum for compound **7**

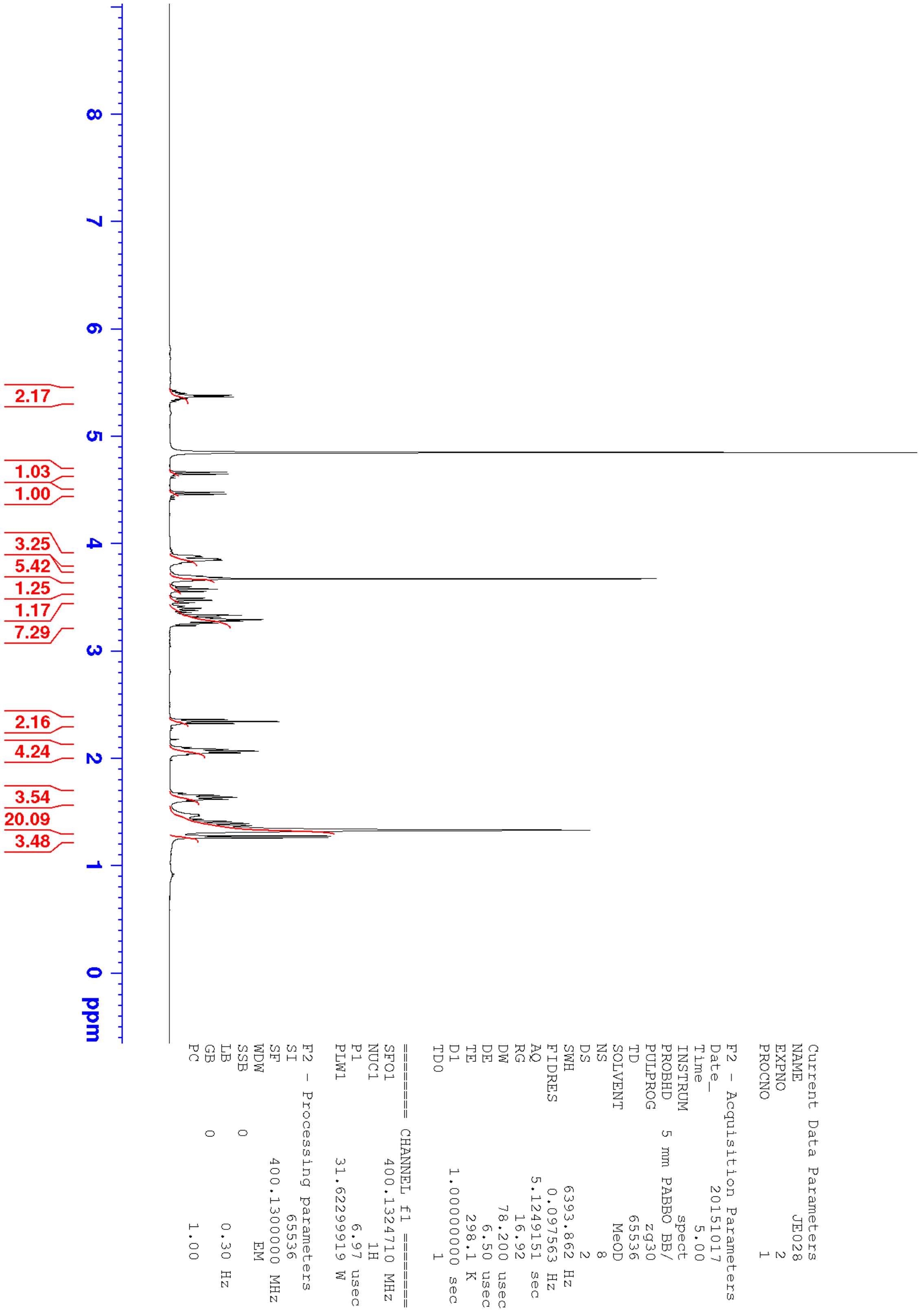

Fig. S7. ^{1}H-NMR spectrum for compound **8**

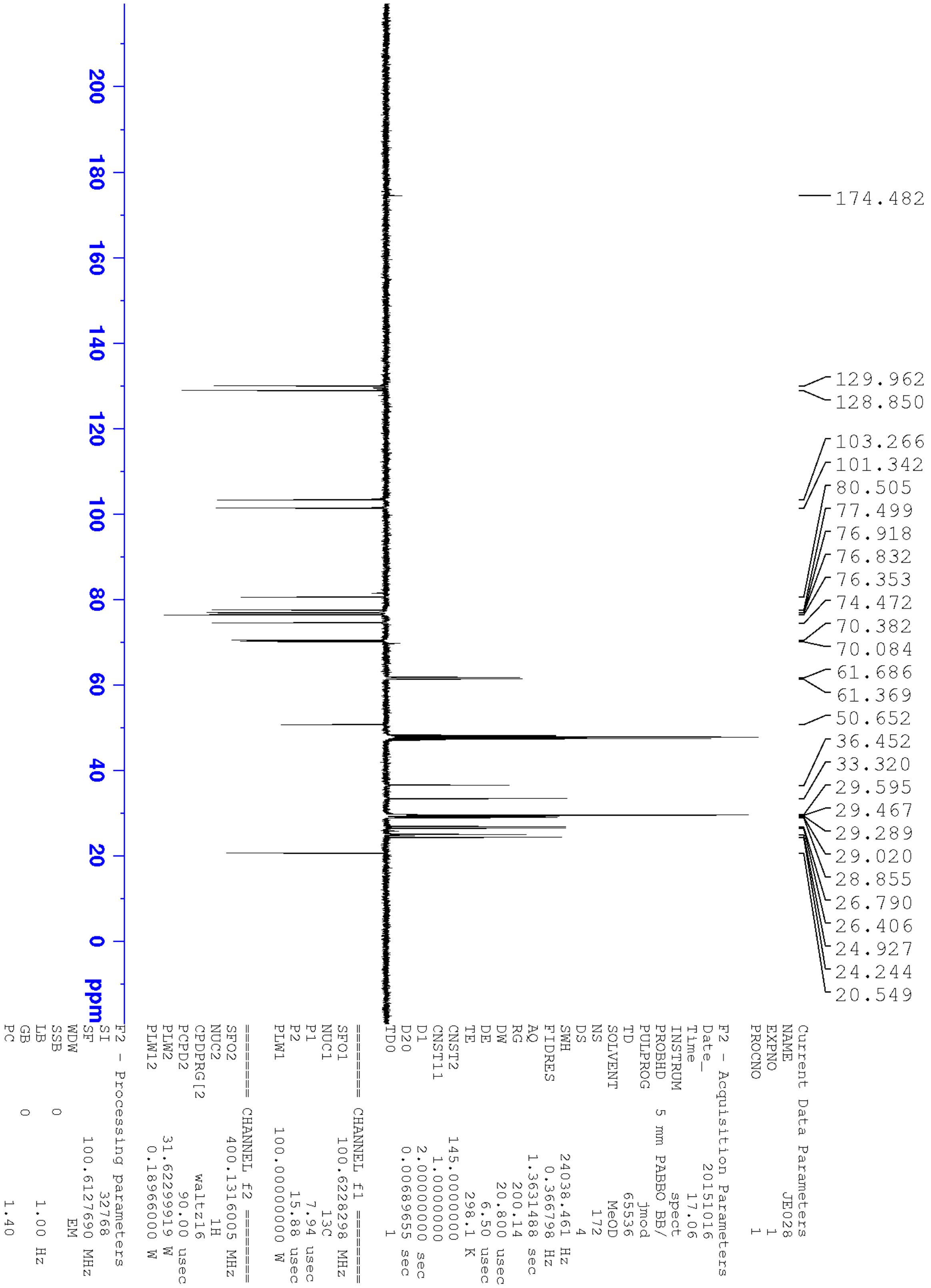

Fig. S8. ^{13}C-NMR spectrum for compound **8**

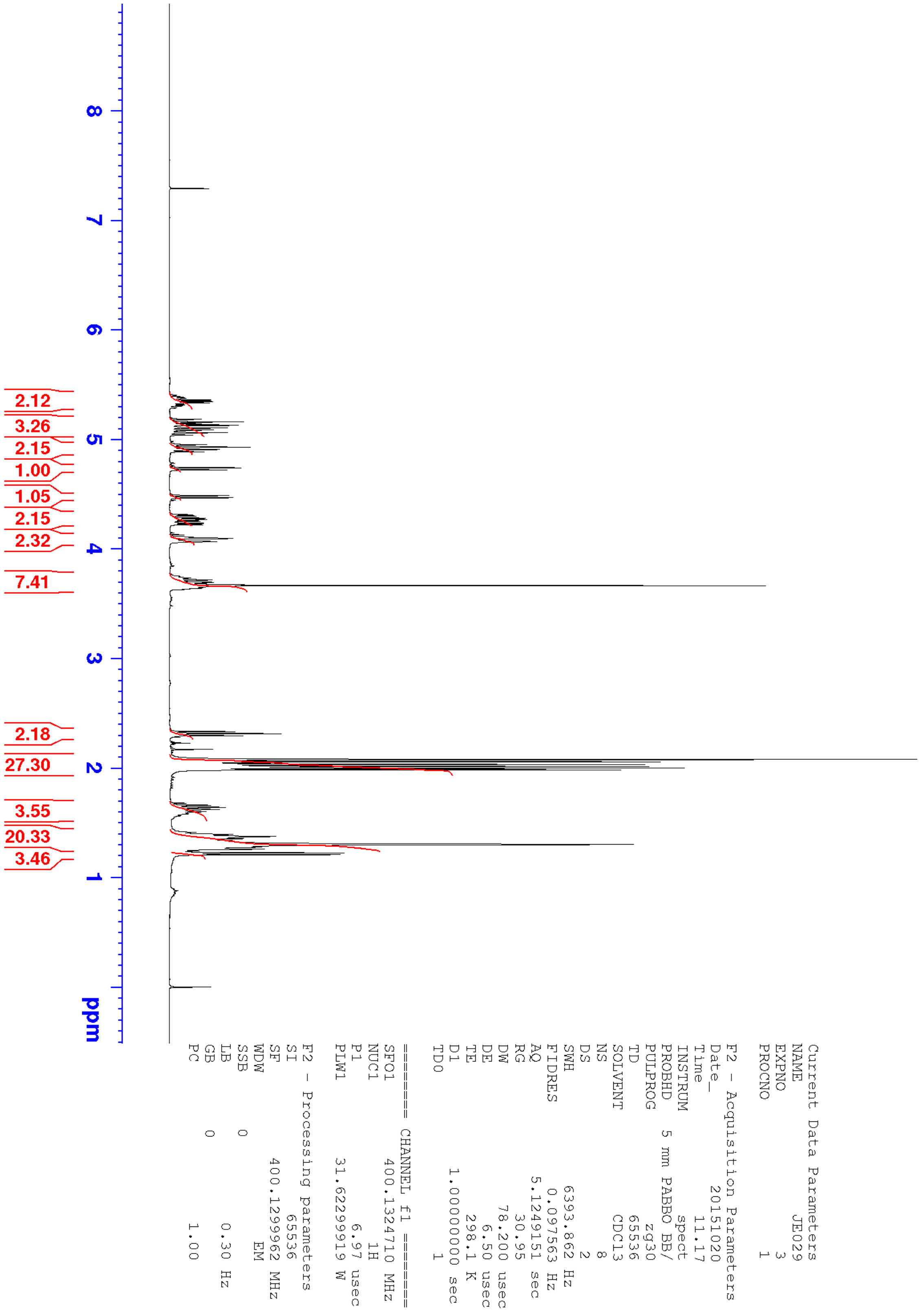

Fig. S9. ^{1}H-NMR spectrum for compound **9**

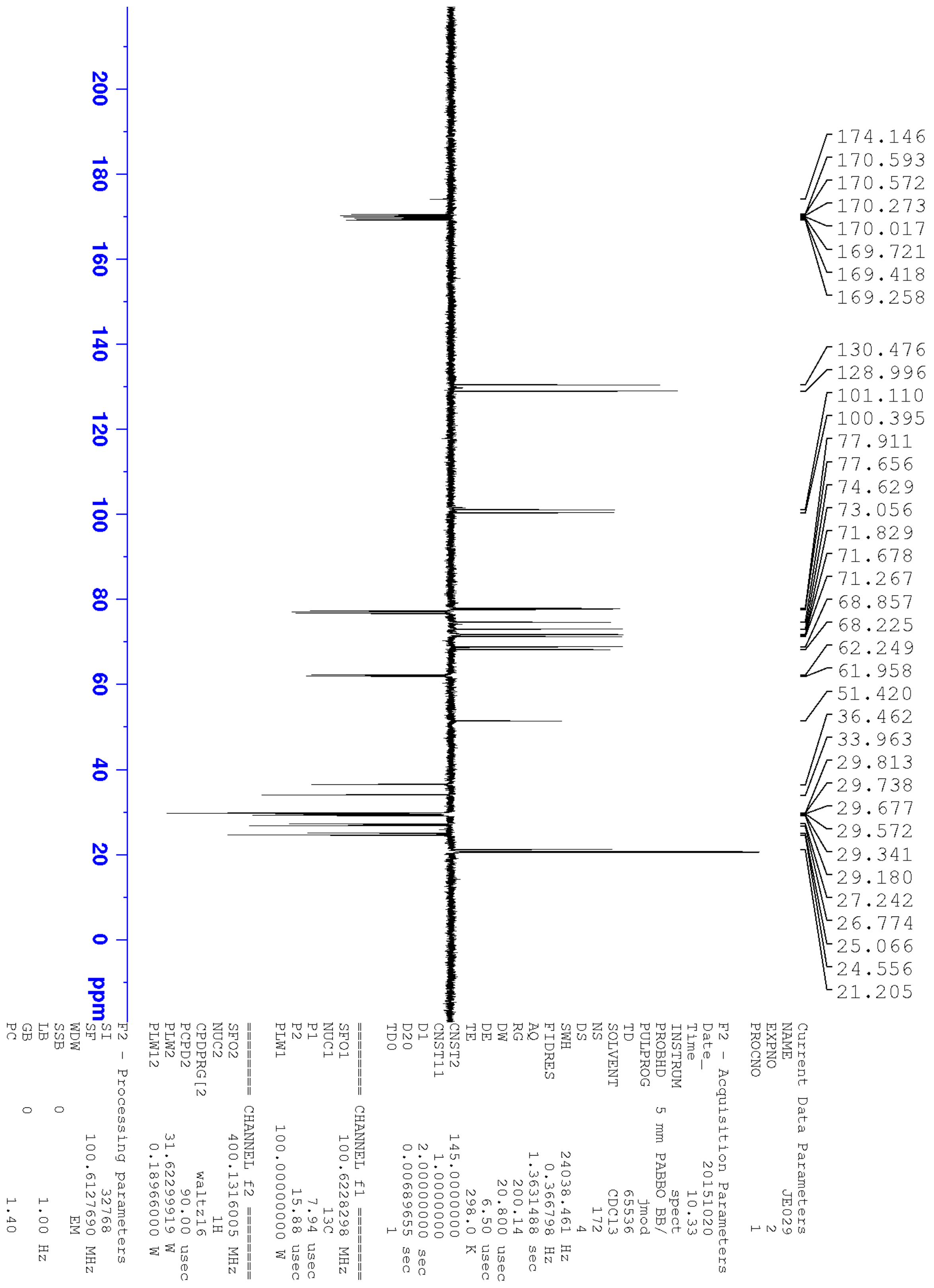

Fig. S10. ^{13}C-NMR spectrum for compound **9**

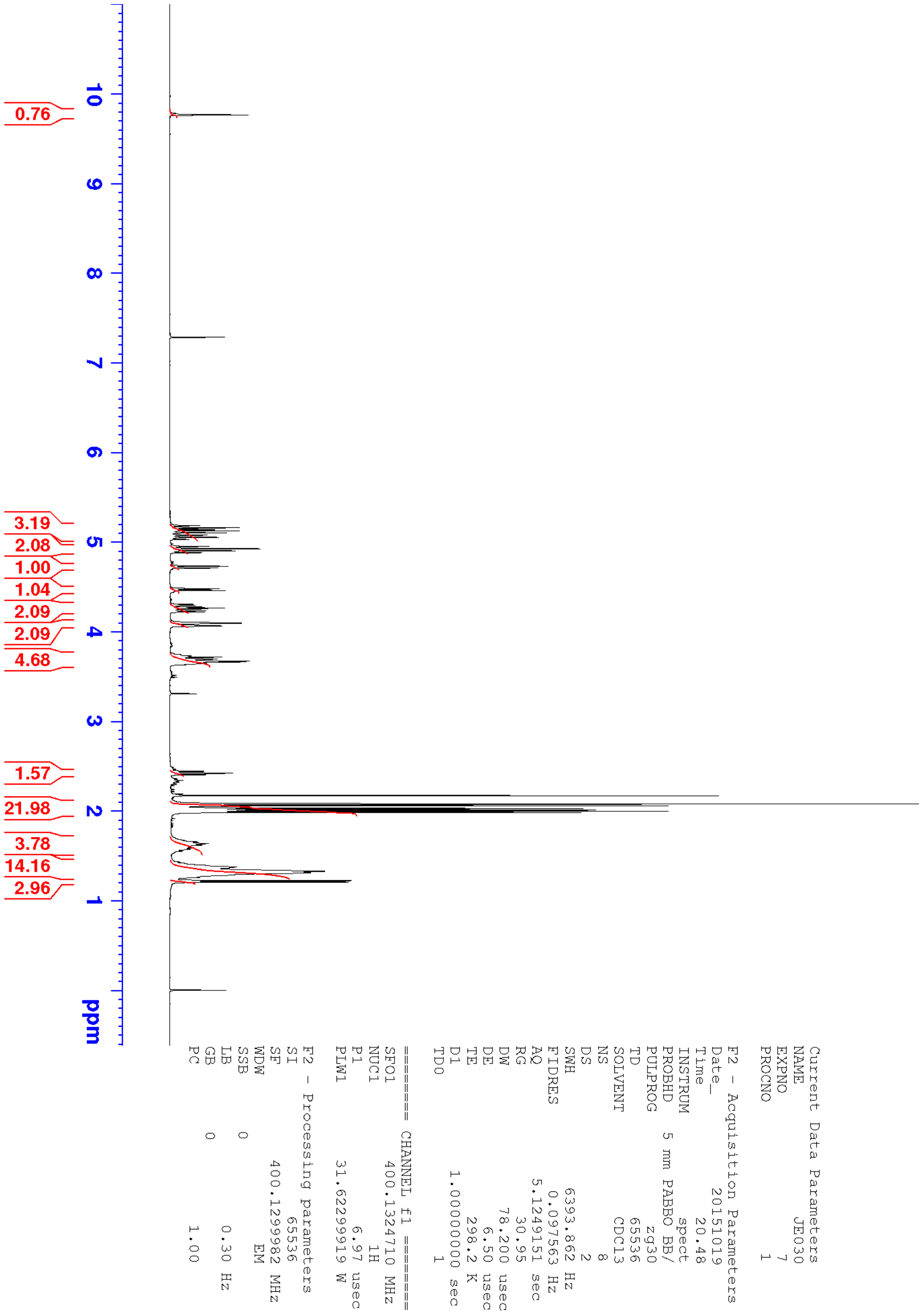

Fig. S11. ^{1}H-NMR spectrum for compound **4**

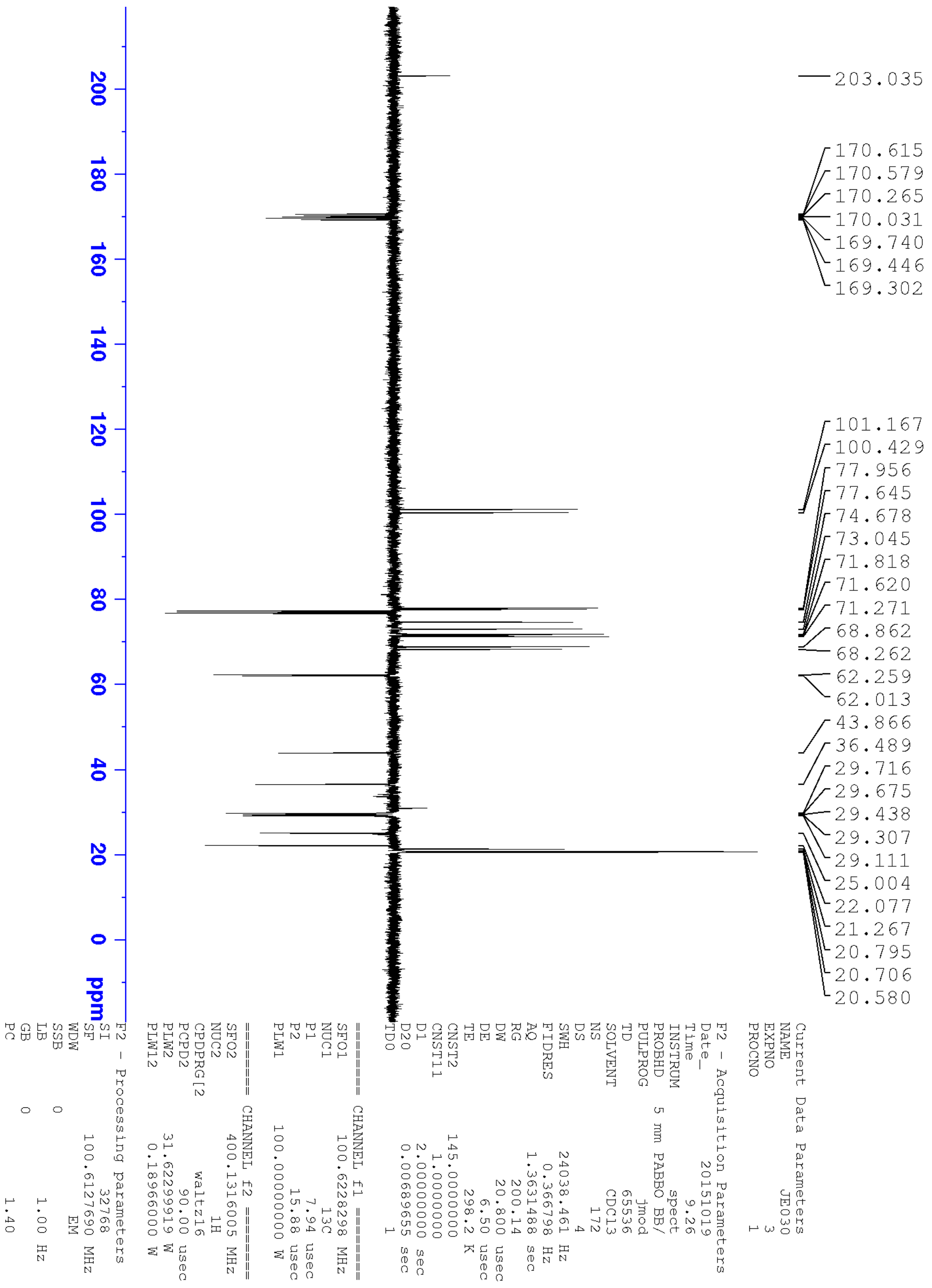

Fig. S12. ^{13}C-NMR spectrum for compound **4**
